# A qualitative study of clinicians’ perspectives on participating in an intrapartum randomised placebo-controlled trial for labour pain relief

**DOI:** 10.1186/s13063-025-09257-z

**Published:** 2025-11-25

**Authors:** Nigel Lee, Belinda Barnett, Sue Kildea, Leonie Callaway, Lauren Kearney, Sania Hellyer, Lena B. Mårtensson

**Affiliations:** 1 School of Nursing Midwifery and Social Work, University of Queensland, Level 3 Chamberlain Building, St Lucia, QLD Australia; 2 Molly Wardaguga Research Centre, College of Nursing & Midwifery, Charles Darwin University, 17 Grevillea Drive, Sadadeen, Alice Springs, 0870 Australia; 3 Women’s and Newborn Services, Royal Brisbane and Women’s Hospital, Level 6, Ned Hanlon Building, Butterfield Street, Herston, QLD 4029 Australia; 4 School of Health Sciences, University of Skovde, Box 408, Skovde, 541 28 Sweden

**Keywords:** Intrapartum research, Midwifery-led, Woman-centred, Equipoise, Pain relief

## Abstract

**Background:**

Supporting intrapartum research through recruitment and intervention delivery and data collection can be challenging for clinical midwives. While there is a need for high-quality evidence of effective care during labour and birth, there are very few midwifery-led randomised controlled trials conducted during this period. Similarly, there is little research on the motivating factors and challenges for clinicians associated with intrapartum research.

**Aim:**

Our aim was to explore the experiences of midwives who participated in a placebo-controlled intrapartum trial.

**Methods:**

Midwives who had participated in a trial investigating the use of sterile water injections for managing abdominal labour pain were invited to participate. Semi-structured interviews were conducted via videoconferencing. Data were transcribed and analysed thematically.

**Results:**

Interviews were conducted with 11 midwives. Three themes were identified: (i) introducing a novel treatment for labour pain; (ii) the conflict of care and placebo and (iii) participating in a clinical trial.

**Conclusions:**

Participants described experiences ranging from the support for an experimental analgesia and expectations of effect, to reservations to participants potentially encountering the placebo treatment. At times the experiences were both positive and negative. Midwives often found the timing of providing trial information and obtaining informed consent challenging. Our findings highlight the challenges encountered by midwives when presenting trial information, establishing equipoise and the timing for these to occur during labour.

**Trial registration:**

The trial is registered at the Australian and New Zealand Clinical Trials Registry (Trial ID: ACTRN12621001036808). Registration date 5/08/2021.

**Supplementary Information:**

The online version contains supplementary material available at 10.1186/s13063-025-09257-z.

## Introduction

Randomised controlled trials (RCT) are considered the ‘gold standard’ of interventional research design [1]. The process of randomising participants to two or more groups assists in balancing observed and unobserved participant characteristics and confounders between groups. This reduces bias and provides a more rigorous approach to determining cause and effect [1]. The use of a placebo further limits bias through blinding researchers, clinicians and participants to the allocated treatment [2].

Despite calls to increase the inclusion of birthing women in research clinical trials into maternity care make up less than 2% of all registered trials and have higher rates of non-completion [3, 4]. The design, complexity, cost and regulatory requirements associated with RCTs, and the common ethical designation of birthing women as a vulnerable population, are recognised barriers to intrapartum research [5, 6]. These factors are also likely to contribute to the relatively small number of RCTs undertaken by midwives. A 2023 scoping review of midwifery-led RCTs in Australia and Aotearoa New Zealand over a 20-year period (2000–2021) identified 50 trials registered on the Australian and New Zealand Clinical Trials Registry and 35 peer-reviewed publications of midwifery RCTs [7]. Supporting strategies for managing women’s pain during labour is an integral part of midwifery intrapartum care, yet just three of the 35 aforementioned RCT publications related to labour analgesia [8–10]. Only one of these RCTs was placebo-controlled [10].

Up to 85% of RCTs fail to recruit to the required sample size and time frame [11]. Trials involving novel treatments and placebo groups are more likely to encounter participant resistance and clinician gatekeeping which impacts on recruitment [12, 13]. Undertaking successful RCTs of non-pharmacological pain management strategies provided by midwives during labour and birth is likely to be particularly challenging given the number of options available to women, including the option of no pain relief. Similarly, obtaining women’s informed consent to RCTs during labour presents a number of practical and ethical challenges related to experience of pain and the potential effect of medication on capacity [10, 14]. While there is a significant body of research regarding participation in RCTs generally and those for chronic pain specifically, few studies have explored the experience of midwives and women’s involvement in midwifery-led placebo RCTs involving analgesia during labour and birth. Acknowledging the potential for this dearth of enquiry into the challenges of intrapartum RCTs to further inhibit or discourage midwifery clinical research, we undertook a qualitative study of midwives and women’s participating in the SATURN trial: a placebo-controlled trial of the use of sterile water injections (SWI) to manage abdominal labour pain [15, 16]. This paper presents the data from the midwives; subsequent publications will focus on women’s experiences. Prior to the SATURN trial, RCTs had only explored the use of SWI for labour back pain; hence, the SATURN trial was unique in this field. The aim of this study was to explore the experiences of midwives who participated in a placebo-controlled intrapartum trial.

## Methods

The SATURN trial was conducted in large metropolitan hospital in Brisbane Australia offering a variety of models of care including birth centre and midwifery group practice. The SATURN trial was a parallel randomised placebo-controlled trial involving 156 women in labour at term requesting pain relief. Women were randomised to either six injections of sterile water or a sodium chloride 0.9% (normal saline) control [16]. To achieve double blinding of both women and midwives, participants were randomised to injections from pharmacy prepared identically labelled ampoules of either sterile water or a normal saline. A topical vapocoolant skin anaesthetic spray was applied to the six injections sites to provide a plausible explanation for any difference in injection pain. Information regarding the trial, in the form of posters and brochures, were available through antenatal clinics. Women could be provided directly with study information from 32 weeks gestation onwards by midwives providing antenatal care or through contact with research midwives. As restrictions in place during the COVID-19 pandemic aimed to limit face to face contact, videoconferencing with research midwives was also an option. Women could provide signed consent prior to labour with the understanding that this did not commit them to participation and consent would be reaffirmed during labour. At study completion, 511 women had provided written consent and 160 randomised. The most common reasons for not participating following antenatal consent were declined to proceed (*n* = 181), not offered by midwife during labour (*n* = 61) and no longer eligible (*n* = 48). Birth (*n* = 20) and epidural use (*n* = 18) accounted for most of the remaining reasons for not participating [16]. Of those participants randomised, 102 provided initial consent during labour and 52 forms were co-signed by midwives who were not research staff.

The use of SWI to manage back pain in labour had been standard care at the research site since 2013 following the participation in a previous SWI RCT [9]. Subsequently, all of the midwives in this study had some familiarity with the procedure in the context of back pain and many considered themselves to be advocates for, and regular uses of, the procedure.

Design: A qualitative study of midwives participating in a RCT.

Participant recruitment: Purposive sampling was employed to recruit participants. Midwives who had experience of recruiting women and/or using SWI as part of the SATURN trial were invited to participate. Midwives were initially approached by the trial research midwife based on their experience of participating in the trial and those expressing interest were provided with trial information. They were then contacted by the researcher conducting the interviews (first author) and invited to provided written consent. The final sample size was determined on analytical and pragmatic judgements that were consistent with the reflexive thematic analysis approach [17].

Data were collected through individual semi-structured interviews based on an interview guide. The interview guide included questions that asked midwives to reflect on their personal midwifery philosophy and approach to supporting women during pregnancy, labour and birth. This provided context for the discussion regarding the experience of incorporating research participation into their practice. Further sections of the interview guide were structured around concepts of interest drawn from the literature on clinical trial participation such as equipoise, placebo use and motivation. Examples are provided in italics.

- Establishing equipoise: *What were your thoughts when you first heard of the trial and the need to randomise women.*
- Using a placebo: *Was the use of a placebo an issue for you? e.g. Did you have any concerns about using a placebo?*
- Challenges in research participation: *What do you think are the main barriers to conducting a trial on the birth suite?*

Interviews were conducted using an internet teleconferencing platform. This approach was preferred due to convenience and to provide a contactless approach in the post-COVID-19 era. Interviews lasted between 20 and 50 min.

Ethical approval was provided by the Hospital Human Resources Committees where the study was conducted and by the university affiliated with the first author.

Individual interviews were audio recorded, transcribed verbatim by a third party, and then verified by the first author. Pseudonyms were allocated to each participant to protect anonymity; other identifying features, including references to individual staff or other persons, were similarly disguised. Data analysis employed a deductive and inductive approach [18]. The concepts of equipoise, placebo use and research engagement provided a guiding structure to the midwives data that was coded using an inductive reflexive analysis as described by Braun and Clarke [19, 20]. The analysis process was iterative; transcripts were read and re-read to identify concepts relevant to the research. Data was imported into NVivo software and coded by the first author. Codes and data were reviewed by the second author and an initial coding structure agreed by consensus. Codes were reviewed against the transcribed text to ensure that they accurately reflected the data entered. A process of collapsing and merging of data codes then occurred before the final thematic structure was confirmed (Table 1). Peer discussions served to challenge perspectives and individual interpretations. Given the differing experiences of maternity care provision of the first (clinical midwife and researcher) and second author (consumer advocate academic), this process enabled sense-checking of assumptions, data interpretation and thematic construction. They contributed to reflexivity and provided a means of analytical triangulation.

**Table 1:** Coding and thematic structure

First level codes	Second level codes (sub themes)	Final thematic structure
• Previous experience of water injections • Need for safe pain relief • Thoughts on SATURN trial • Uncertainty of effect	• It should work • Developing equipoise	Introducing a novel treatment for labour pain
• Midwives inflicting pain • Placebo or deception • Experimenting on women • Choice or no choice • Women’s reactions to placebo observed by midwives	• Professional conflict • Supporting women’s autonomy	The conflict of care and placebo
• Engaging with research • Experience of recruitment • Experience of randomisation • Timing consent during labour • Women’s reactions to recruitment observed by midwives	• Responsibility and leadership • Antenatal versus intrapartum recruitment • Finding the ‘sweet spot’	Participating in a clinical trial

## Results

Interviews were conducted between October and November 2023. Of the 15 midwives initially expressing interest, 11 were interviewed. All participants were female, aged between 25 and 55 years with between 3 and 33 years of experience (Table 2).

**Table 2:** Participant demographics

Participant data	*N* (%)
Age	*N* = 11
30 years or less	5 (45)
31 years or more	6 (55)
Initial midwifery training	
Hospital certificate	2 (18)
University degree	9 (82)
Years of experience	
10 years or less	6 (55)
11–20 years	2 (18)
21 years or more	3 (27)

Through data analysis, three main themes were identified: (i) introducing a novel treatment for labour pain; (ii) the conflict of care and placebo and (iii) participating in a clinical trial. Quotes from midwives that are illustrative of the themes are provided. Some quotes have been edited to maintain focus on the topic under discussion. The format […] indicates where this has occurred.

### Introducing a novel treatment for labour pain

This theme explored the reactions of midwives to a novel idea of using SWI to relieve abdominal labour pain. Midwives were introduced to the concept of using SWI for abdominal pain and the procedure for administration of the injections via an online training program specifically designed for the study and face-to-face in-services prior to the commencement of recruitment. The procedure involved six intradermal injections to predetermined sites across the abdomen of the labouring woman [15]. Individual reactions to new treatment options highlighted degrees of acceptance through to caution. Midwives previous experience provided some with a rationale for accepting the potential of SWI for managing abdominal labour pain.

*I thought if we are getting good results for sterile water injections with back pain, why wouldn’t it work for abdominal pain? It made sense to me that it could and would work.* (Veronica).

*[…] I’ve obviously done many sterile water injections into the back. So hearing about it, I was like, oh, OK, this is cool. This is something different.* (Letitia).

Other midwives were more circumspect, expressing questions over how the technique might be effective for abdominal labour pain. One midwife raised the question that if the injections were effective in this manner, then this would have been researched earlier:

*I thought how’s this going to work? […] surely it can’t be that effective because surely we would have done It already.* (Cassandra).

Some midwives expressed reservations about the effectiveness, being unsure of the potential physiological mechanisms, but being curious.

*Really, I wasn’t certain that there was any kind of, like, nerve thing going on in the front that would resonate with me in the same way as the back. So. I wasn’t sure.* (Vanessa).

Some also considered potential benefits of the use of abdominal pain SWI in their initial reactions. Other midwives acknowledged the possibility of expanding non-pharmacological analgesia options for labouring women while still expressing some questions and reservations of mode of effect.

*I knew that there was a potential for this to be a game changer for woman who may just ordinarily select an epidural or a narcotic of some sort. But I was not aware of the nerve pathway. Or how that would actually influence the, the level of pain that woman may experience or not experience? I didn’t even contemplate how that could actually, you know, all the injections in the belly, how that would work. But I was intrigued.* (Kelly).

For midwives in this study, the idea of using SWI to relieve abdominal labour pain had to resonate with them on both a clinical and physiological level while also presenting a level of uncertainty to justify conducting a trial.

### The conflict of care and placebo

As outlined in the Methods section, the SATURN trial involved half of the participants receiving an injection of normal saline, which was designed to have little if any effect on labour pain [15]. Given that an important aspect of midwifery and woman-centred care is open and honest communication, the concept of a placebo did introduce some contradiction in their attitude to the trial and approaching women to participate. A number of midwives expressed mixed feelings when confronted with the desire to provide effective care, including pain relief, and the potential of providing an ineffective placebo and the need for research.

*But you did feel bad, especially coming from the place of the midwife where all you want to do is help. You’re like oh am I really helping by giving this, but then we can’t do research and get good results without it. So, it was sort of like two minds in one.* (Letitia).

The fact that allocation to the placebo was likely to result in analgesic failure, seen as the antithesis of normal intrapartum care, led to conflicting ideas of benefit versus potential harm from continued pain.

*A little bit conflicted at times. […] am I doing the right thing by offering this? If it’s going to hurt them, but they’re still not going to get that pain relief that they were after. The placebo issue was a little bit of a barrier.* (Letitia).

Other midwives raised the issue of placebos and ‘experimenting’ on women as a factor that caused them personal and professional discomfort. This was particularly concerning for one midwife who worked with Indigenous women. The midwife recounted how discussing the placebo aspect of the trial appeared to trigger feelings of distrust and anxiety, likely due to the well-known historical abuse of the Indigenous population in medical research [21].

*Women feeling like they were experiments I hate, I hated like just a matter of whether you’re lucky or not if you get the placebo or the real deal. I’m sure I had at least four women say I’m not going to be one of your experiments. But also I think we have to keep in mind that that was all through [Indigenous women’s clinic). You know that there was stigma associated with First Nations women being part of a trial. I think some women, it was definite I could feel there was tension when I brought it up and. I didn’t want that.* (Cassandra).

Other midwives discussed similar encounters with non-Indigenous women who questioned the need for experimentation with a placebo and the need for randomisation.

*It was an issue with me. Some of the women were being miffed about the whole thing. They’re like, well, if we’re gonna do this trial, why can’t we be guaranteed to have the actual [water] injection itself.* (Chloe).

There were other midwives who took a more circumspect approach considering that the use of a placebo was an integral part of the trial process. Furthermore, that the decision to participate, or not, was a further expression of the birthing woman’s autonomy.

*We don’t know if you’re getting water. We don’t know if you’re getting saline, but it’s still worth a shot because if it works, great. If it doesn’t again, we just try something else. So I personally didn’t have any concerns with that because to me, that’s research I feel.* (Veronica).

*That wasn’t a barrier for me, because it was all about the woman’s choice she understood that was either going to be the right stuff or not, so if they were happy with their decision, then I was happy to continue trialing it.* (Lindsey).

Midwives also discussed ways of talking to women about the use of placebos though also acknowledged the challengers of such conversations.

*Initially it was* [hard]. *But I think over time I found conversation that helped work through that. I think it was just basically being honest. […] making this research real. So that we could start doing something that would also contribute to women’s ongoing list of pain relief.* (Vanessa).

Some midwives discussed the reactions of the labouring woman’s family and support persons to the concept of receiving a placebo and how this could influence the woman’s decision to participate or not.

*One lady who was really, really keen, and she hadn’t been consented yet, and her mom was with her, and she was reading through the consent form, and her mom talked her out of it because her mom [said], “it might not even work like, you may as well get an epidural”.* (Brooke).

The midwives data illustrates the often individualised struggles with the concept of placebo use and how, for some, this challenged their ideas of woman-centred care. While others reflected on the concepts of women’s agency and choice to rationalise the presentation of the placebo aspect to women as an important component of research.

### Participating in a clinical trial

The strategies to promote the SATURN trial and encourage birthing women to consider participation included pamphlets and posters positioned in key areas such as antenatal clinics and obstetric review centres. Consent was then either sought by the research midwife antenatally or by a birth suite/centre midwife during labour. For those women who did consent antenatally, consent was reconfirmed during labour to ensure they were still interested in participating. Midwives commented on having a preference for the staged approach to information and consent. This entailed providing women with copies of the study information and then reinforcing that they can discuss details and answer questions at a future appointment.

*The Saturn trial I personally found it easy just to give them a handout and say take this home, read it, bring it back for the next appointment I can explain it. and we can consent you if you’re interested.* (Veronica).

Of the 511 women consented to the trial, 351 did not proceed to randomisation during labour. A number of midwives commented that, for a variety of reasons, the re-establishment of consent during labour proved challenging.

*I feel like the recruiting part is kind of the easy part. The converting* [reconfirming consent] *part in labor is the hard part.* (Brooke).

Some midwives discussed the range of factors that would need to align for participation in the trial to proceed. These elements ranged from how women were personally coping with labour to overall progress in labour.

*I think the recruitment is, you know, getting people on board to say, oh, yeah, yeah, I’ll be part of that trial. But actually, on the day I think it’s difficult depending on how they’re feeling, how the labours going, what state of labour they present? There’s like lots of different factors that could affect them actually going through with the trial.* (Andrea).

Other midwives cited how rapidly situations could change during labour and they sometimes struggled to find the ideal moment to begin the conversation about the trial. There was acknowledgement that while this ‘sweet spot’ was difficult to define it was advantageous to discuss the trial before the degree of pain reached the point where only pharmacological analgesia would be considered a viable option.

*Then it kind of seems to escalate really quickly from a point, and then it’s too late. So trying to get in that like really really fine, sweet spot of when to do it. I’ve I found that was quite hard, even if you try to, you know, you talk about all the options at the start of the labor.* (Veronica).

*…you’ve gotta offer it about this point because if you leave it too late, they’re gonna want an epidural. So I think we all got pretty good at knowing exactly when to offer it.* (Chloe).

There was also recognition that at times, with the general activity of the birth suite, midwives may have not remembered to discuss the trial with eligible women. In these situations, some midwives found the prompts provided by the research midwives and trial champions assisted with the occurrence and timing of the discussion.

*When we were in birth suite the research assistants would knock on the door. Hey, have you had a chance to chat about [the trial]? Because we might have forgotten to talk to them at that point about the research study. So it was good to get reminded.* (Veronica).

Midwives also discussed a number of issues that influenced engagement with research and recruitment more broadly. Many midwives in the study acknowledged the profession had a responsibility to support research.

*Honestly, I think it just comes down to us. […] the midwives on the floor taking that responsibility to be involved in research.* (Veronica).

Though the often parttime nature of a predominantly female workforce with other caring responsibilities was considered by some midwives to be a factor contributing to limited engagement with research.

*I think we have a lot of part time staff, you know that do two shifts a week that really just want to come to work and go home. […] they’ve got families. They’re not really interested doing anything else.* (Andrea).

Leadership and role modelling were also considered to be important. A number of midwives observed that leadership support emphasised the standing of research generally in the clinical environment and, in doing so, encouraged broader engagement with trail processes such as recruitment.

*Yeah, […] If the junior midwives see that the seniors are proactive motivated and encouraging of research I think the juniors would be more inclined to follow.* (Veronica).

## Discussion

While the findings of this study offer a number of insights into the perspectives and attitudes towards aspects of intrapartum research engagement, the most striking outcome was the midwives’ reactions to the use of a placebo arm. The use of SWI for labour back pain is well established; however, the use for abdominal labour pain was novel and experimental [15]. As such the use of a placebo arm, in which analgesic failure was expected, was an essential design component to explore analgesic efficacy while limiting bias. The views of midwives towards the use of placebo did vary. While several were content to support the woman’s decision, for others the prospect of providing an intervention, essentially in response to a woman’s request for analgesia, involving an equal chance of ‘failure’ conflicted with their sense of the midwife’s role in labour pain management. For other midwives, the conflict was underpinned by their observed reactions of some women objecting to the chance of placebo as a reason to decline participation.

Midwives’ initial reactions to the use of SWI for abdominal labour pain reflected a continuum from curiosity to scepticism. While most were familiar with SWI for back pain, extending the method to abdominal pain prompted uncertainty about mechanism and efficacy. The process of engaging with the trial required midwives to reconcile their professional identity as advocates for women with their role in generating evidence through the randomised design. These findings support earlier work suggesting that clinical equipoise is not only a theoretical principle but a lived, negotiated stance for practitioners [22]. While a number of definitions of equipoise exist, it generally refers to genuine uncertainty about the effects and benefits of the treatments being compared in a trial, i.e. that an agreed sense of uncertainty exits amongst clinicians or experts [23]. A state of equipoise is considered essential for randomisation, particularly to placebo control groups, to be ethically sound [24]. Studies by a number of researchers have considered the issue of equipoise amongst clinicians and how this influences trial engagement. For clinicians, a clear understanding of the trial in question without presumption of likely outcomes is needed to convey equipoise to participants [22]. In the present study, equipoise was sustained when midwives could justify uncertainty scientifically, but strained when placebo allocation conflicted with their philosophy of woman-centred care.

Data provided by midwives in this study reflected the two recruitment pathways used in the trial. Most women provided informed consent antenatally and verbally reconfirmed consent during labour. Alternatively, some women provided consent for the first time during labour. Previous studies of the challengers of recruitment to intrapartum trials have also concluded that a two-stage approach to participant recruitment is ethically ideal [25]. However, offering trial information antenatally also presents a number of issues. Providing pregnant women with detailed information of potential but otherwise rare risks to themselves or their foetus may cause unnecessary anxiety and not be applicable to most until the event occurs during labour [26]. Though assumptions regarding how potential participants may respond to information regarding risk may also lead to ‘gatekeeping’ and impinge upon the ethical right to self-determination [25]. Furthermore, similar to findings from a study of ethical challengers of mental health research midwives in this study identified moral and emotional conflicts when balancing patient welfare with research objectives [27]. Unlike prior work focusing on logistical barriers [28], our findings emphasise ethical and relational dimensions—particularly the alignment (or misalignment) between research protocols and woman-centred care philosophies. The process of providing trial information also involves considerable resources. Some studies have reported that while online or remotely provided trial information was more economical, face to face was more costly though resulted in higher numbers of conversions to participant consent and enrolment [29].

Midwives commented on the challenges of providing consent during labour and finding the ‘sweet spot’ when discussion of the trial was deemed suitable. The limited research on obtaining consent from women during labour and birth highlights two issues of capacity and trust. As reflected in the data provided by midwives, clinicians may assess women’s capacity to consent during labour, often based on observations of pain and/or progress. A retrospective observational study of clinician’s assessment of women’s capacity during labour reported considerable heterogeneity between assessors’ judgement of lack of capacity [30]. Other authors have commented that restricting participation based on ‘cut-off’ labour progression criteria (e.g. 6 cm cervical dilation) is ethically unsound, rather assessments should be made on an individual basis [31]. Researchers have also suggested that as a birthing woman’s capacity to take in new information declines during labour the reliance on trust in their clinician increases [32]. As such, the unpredictable nature of labour and the timing of consent can create some discordance between what is detailed within the trial protocol and what actually occurs during intrapartum recruitment [33]. However, overall midwives found seeking or reconfirming consent in labour was appropriate.

The findings of this study suggested broader organisational and leadership factors shaped midwives’ engagement in research. Participants described variation in institutional support, noting that visible endorsement from senior staff encouraged participation, whereas competing clinical demands and limited research infrastructure constrained it. These findings are consistent with international literature identifying leadership, workload and perceived relevance as determinants of clinicians’ research engagement [34]. Previous studies have highlighted that while midwives generally support research engagement a lack of support, resources training and experience were barriers to participation [35]. An Organisation for Economic Co-operation and Development report describes research engagement as a complex social process including interaction between resources, structures and processes, and incentives which are facilitated by leadership [36]. Leadership structures that were not conducive to research result in low prioritisation of research engagement amongst clinicians potentially to the point of discouraging attempts to change clinical practice [37]. Role modelling was also viewed as a means of encouraging research engaging particularly with early career clinicians. Knowledge brokers have been introduced in some health systems to facilitate the transition from research into practice and to also develop capacity in terms of research engagement and production [38]. However, research into the most effective and efficient ways to engage clinicians generally and midwives specifically in supporting research remains elusive [35, 39]. The experiences reported here suggest that promoting midwives’ involvement in research requires not only leadership advocacy but also systemic strategies—such as allocating dedicated research time and integrating research competencies into professional development frameworks.

The strengths of our study lie in presenting data from midwives involved the relatively rare occurrence of a midwifery-led placebo-controlled intrapartum trial. As with many qualitative studies, the results provide insights into participants’ personal experiences of the phenomenon in question and are not intended to be generalisable to other populations. The sample comprised 11 midwives from a single metropolitan site, which may limit transferability to other settings. Participants were largely familiar with SWI for back pain, which may have shaped their openness to the intervention and introduced a positive bias. Additionally, the first author’s multiple roles as midwife, researcher, interviewer and primary coder may have influenced interpretation; however, reflexive journaling, peer debriefing and co-author review were employed to enhance rigour. Furthermore, there is the potential for recall bias as participants’ recollections and interpretations of personal interactions may have changed in the weeks between the experience and the point in time in which the interviews were conducted.

The findings of our study hold several implications for strengthening midwifery engagement in clinical trials. First, education and training should address both ethical and communicative aspects of research participation. Structured workshops that equip midwives to discuss equipoise, placebo use and informed consent could enhance confidence and reduce moral tension. Second, institutional leadership plays a pivotal role: visible endorsement from senior midwives and the inclusion of ‘research champions’ within maternity units can normalise research participation and counteract perceptions that it conflicts with woman-centred care. Third, policy and funding frameworks should recognise the additional time and relational work required for ethical recruitment in intrapartum settings. Resourcing antenatal education, staged consent processes and dedicated research midwife positions should be viewed as essential trial components rather than ancillary costs.

For future research, these findings suggest a need to examine strategies for integrating research into routine maternity care, including evaluating models of leadership, mentorship and organisational culture that effectively support midwifery research engagement. Comparative studies across diverse settings could identify context-specific enablers and barriers. Moreover, exploring women’s perspectives on consent and placebo use, as planned in the companion paper, will provide a more comprehensive understanding of relational ethics in intrapartum RCTs.

## Conclusion

This study provides insights into the important and under-researched issues of seeking consent for participation in intrapartum placebo RCTs. Our findings highlight the challenges encountered by midwives when presenting or considering trial information, establishing equipoise and the timing for these to occur during labour. Our findings support the ethical utility of the staged approach to obtaining intrapartum consent, acknowledging that the time between the woman’s initial introduction to the trial information and the final confirmation of consent can be variable. If using this approach, then the cost of antenatal education, information and consenting, alongside support for research midwives in the birthing suite, must be factored into trial budgets.

## Supplementary Information

Supplementary Material 1.

Supplementary Material 2.

## Data Availability

Due to the nature of this qualitative study, interview participants were assured that raw data would remain confidential and would not be shared.

## Abbreviations

RCT: Randomised controlled trial
SWI: Sterile water injections
