# Supplementary material for: A qualitative study of clinicians’ perspectives on participating in an intrapartum randomised placebo-controlled trial for labour pain relief
Source: Trials. 2025 Nov 25;26:547. doi: 10.1186/s13063-025-09257-z (PMC12648800; doi:10.1186/s13063-025-09257-z)
Supplement: Supplementary file 1 — Supplementary Material 1. [file 13063_2025_9257_MOESM1_ESM.docx]

Supplementary file 1

Lead author reflexivity statement

As Chief Investigator on the SATURN trial and this qualitative study I conducted all the interviews and reviewed the transcriptions. Consistent with the Braun and Clarke approach to reflexive thematic analysis I took the lead role in coding, data analysis and theme construction. The coding and thematic structure was then reviewed and discussed with other authors

I am a midwife with 30 years clinical experience in labour and birth care. I am a clinical trials and multi-methods researcher having undertaken and published quantitative and qualitative research into labour pain management and strategies to support physiological birth. My viewpoint is centred in the idea that birth interventions should be evidence based and congruent with the midwifery philosophy of woman-centred care.
